# Supplementary material for: Adaptation and validation of HIV/AIDS module for university students in Larkana, Sindh, Pakistan
Source: PLOS Glob Public Health. 2026 Mar 25;6(3):e0006159. doi: 10.1371/journal.pgph.0006159 (PMC13016276; doi:10.1371/journal.pgph.0006159)
Supplement: Attachment — Submitted filename: Rebuttle Letter .docx [file pgph.0006159.s002.docx]

- Reviewer #1: I have read the referred article with keen interest. The information is interesting and innovative; conclusion section is interesting and authors can improve it further. I am recommending authors to do a little more work and add latest literate to support the study. The authors need to improve results section. The level of English is good and smooth, e.g., the language standard, specifically the grammar, of sufficient quality to meet scientific merit for publication. However, I suggest authors to double check for language quality. Describe scientific contribution of the study to the existing body of knowledge. I endorse this manuscript after minor revision as suggested. The topic is interesting and worthy of attention. The methodology is adequate and the conclusions are consistent with the reported data. The manuscript can be improved by expanding the references and citing some recently published articles on this topic.

  Authors should consider the following recommendations
- I recommend further improving the references by citing some of these recent studies on the topic:
- Anxiety and Social Functioning among People Living with HIV/AIDS (PLWHA) in Pakistan: A Cross-Sectional Study
- Opportunities and Challenges of Generative AI in Pakistani Higher Education: A Qualitative Study on Student Perspectives in Learning, Integrity, and Innovation
- Assessing the Feasibility of Metacognitive Training for Patients with Schizophrenia in Pakistan: A Randomized Controlled Trial
- Feasibility Evaluation of the Listen Protect Connect (LPC) Intervention for School Students in Pakistan: A Cluster Randomized Controlled Trial
- A hindrance to proper health care: psychometric development and validation of opiophobia questionnaire among doctors in Pakistan

Response: We thank the reviewer for suggesting additional references. We have carefully reviewed each recommendation and incorporated two highly relevant studies:

1. The study on anxiety and social functioning among PLWHA in Pakistan has been cited in our Introduction to strengthen the rationale for targeted HIV/AIDS education, highlighting the psychosocial burden faced by affected individuals in our context.
2. The feasibility evaluation of the LPC intervention for school students in Pakistan has been cited in our Discussion to situate our validation methodology within the broader landscape of educational intervention research in Pakistani settings.

Regarding the other three suggested references:

- The study on generative AI in higher education addresses technology integration rather than health education content validation
- The metacognitive training study for schizophrenia patients focuses on a different clinical population and intervention type
- The opiophobia questionnaire study examines physician prescribing behaviors rather than student health education

While these are valuable contributions to Pakistani health research, they fall outside the specific scope of our HIV/AIDS educational module validation study. We have instead ensured our reference list includes the most recent and directly relevant literature on HIV/AIDS education, validation methodologies, and youth health interventions in similar contexts.

Reviewer #2: The manuscript addresses an important public health topic and is well-organized, with a clear. The study design, methodology, and analysis are appropriate, and the findings are clearly presented. Overall, the manuscript is of good quality, provides valuable insights, and is acceptable for publication. I have no further comments.

Response: we highly appreciate your feedback
