# Supplementary material for: Adaptation and validation of HIV/AIDS module for university students in Larkana, Sindh, Pakistan
Source: PLOS Glob Public Health. 2026 Mar 25;6(3):e0006159. doi: 10.1371/journal.pgph.0006159 (PMC13016276; doi:10.1371/journal.pgph.0006159)
Supplement: Attachment — Submitted filename: Rebuttal Letter_Updated.docx [file pgph.0006159.s003.docx]

# **Point-by-Point Response to Reviewers**

**Manuscript Title:** Adaptation and Validation of HIV/AIDS module for university students in Larkana, Sindh, Pakistan

We sincerely thank the reviewers for their thoughtful comments and constructive feedback. We have carefully addressed each concern and made corresponding revisions to the manuscript. Below is our detailed point-by-point response to each reviewer's comments.

## **Reviewer #3**

**Comment 1: Consistency of terminology**

**Response:** We have corrected the title from HIV/AIDs to HIV/AIDS throughout the manuscript to maintain consistency with standard medical terminology.

**Changes Made:** Title has been revised using track changes to read "HIV/AIDS" (all caps for "AIDS").

**Comment 2: Person-centred language**

**Response:** We have revised the introduction to use more dignified and person-centered terminology that respects individuals living with HIV/AIDS.

**Changes Made:**

• Revised from 'HIV has been transmitted to' to 'individuals worldwide are living with or have been affected by HIV'

• Changed 'transmitted among youngsters' to 'new HIV infections occur among young people'

**Comment 3: Asia-Pacific evidence**

**Response:** Added evidence from regional studies and Asia-Pacific data to better situate our work within the regional context.

**Comment 4: Rationale for Larkana City**

**Response:** Added new subsection 'Study Site and Rationale' under Methods describing: HIV prevalence in Sindh/Larkana, high-risk factors, university presence, and limited existing HIV education programs.

**Comment 5: Citation 5 verification**

**Response:** We apologise for this citation error. Citation 5 (Siddiqui et al., 2009) does not discuss delayed marriage as a risk factor.

**Changes Made:**

1. Removed 'delayed marriages' from the list or provided a proper citation

2. Verified all other citations for accuracy

## **Reviewer #4**

**Response:** We thank Reviewer #4 for confirming that previous concerns have been adequately addressed, including psychosocial aspects and appropriate references.

## **Reviewer #5**

**Concern 1: Missing Jordan et al. citation**

**Response:** The Jordan et al. reference has been added to the reference list with proper in-text citation formatting.

**Concern 2: Ethics approval from Malaysia**

**Response:** Added explanation: The first author was a PhD student at Universiti Kebangsaan Malaysia during data collection. UKM policy requires ethics approval for all student research regardless of location. Both UKM (supervising institution) and SMBBMU (implementing institution) approvals ensured compliance with institutional and local ethical standards.

**Concern 3: Sample size justification**

**Response:** Added new subsection 'Sample Size Determination' explaining that 48 students follows Hertzog (2008) and Taherdoost (2016) recommendations for face validity (10-20 participants per item category). For 8 content domains, 40-48 participants was appropriate. For content validity, 11 experts exceeded Lynn's (1986) recommended 6-10 experts. Face and content validity focus on expert judgment quality, not population representativeness.

**Concern 4: Citation issues**

**4a. Davis (1992):**

Removed incorrect Davis (1992) reference. Corrected to cite Lynn (1986) and Polit & Beck (2006).

**4b. Lynn (1986) age:**

Lynn (1986) remains the foundational work for CVI methodology, still widely cited. Added recent validation studies (Marzuki et al., 2018; Lau et al., 2018) to demonstrate contemporary application while retaining the methodological gold standard.

**4c. 'et al.' usage:**

Corrected all instances of 'Polit et al.' to 'Polit & Beck' throughout (only 2 authors, should not use 'et al.').

**Concern 5: Data collection and statistical analysis**

**Response:** Added new subsection 'Data Collection and Statistical Analysis' detailing: 8-week data collection period, software used (Excel 2019, SPSS 26.0, R 4.2.1), statistical procedures (Fleiss' Kappa, I-CVI, S-CVI/Ave), and interpretation criteria.

**Concern 6: QUEST and study site**

**Response:** Added new subsection 'Study Setting and Site Selection' explaining:

• QUEST = Quaid-e-Awam University of Engineering, Science, and Technology

• Student distribution: 24 from SMBBMU medical/allied health (50%), 24 from QUEST engineering/technology (50%)

• Rationale for site selection: HIV prevalence, diverse student population, limited existing education

All changes have been made using track changes in the manuscript for easy identification and review.

We believe these revisions have substantially strengthened the manuscript and addressed all reviewer concerns comprehensively. We are grateful for the reviewers' expertise and constructive feedback.
