# Supplementary material for: Adaptation and validation of HIV/AIDS module for university students in Larkana, Sindh, Pakistan
Source: PLOS Glob Public Health. 2026 Mar 25;6(3):e0006159. doi: 10.1371/journal.pgph.0006159 (PMC13016276; doi:10.1371/journal.pgph.0006159)
Supplement: Attachment — Submitted filename: Response Letter .docx [file pgph.0006159.s004.docx]

Reviewer #3: There are some statements that need proper citation to ensure it reliability. Likewise, the authors need to double-check whether all citations do reflect the argument that they are presenting. In particular, there is one statement regarding the effect of delayed marriages on the STI vulnerability of young people but the original publication that is being cited has not referred to this at all - I wonder if this is a mistake in terms of citation, and if yes, I suggest looking for proper citation.

Response: Thank you for pointing this out. We have made the correction and have rechecked all the references.
